# Verification of Markerless Gait Analysis: Multi-Camera and Single-Camera Approaches in Comparison to Marker-Based Gait Analysis

**DOI:** 10.3390/medicina62020418

**Published:** 2026-02-22

**Authors:** Yong Seok Park, Yeon Woo Yu, Hari Cha, Joon Seok Lee, Chan Yoon, Byung-Hoon Kim, Jae Hyeon Park, Ki-Kwang Lee

**Affiliations:** 1 Department of Sports Science, Kookmin University, Seoul 02707, Republic of Korea; pys9610@gmail.com (Y.S.P.); ka4026@kookmin.ac.kr (Y.W.Y.); 2 EverEx, Seoul 06628, Republic of Korea; charlie@everex.co.kr (H.C.); james@everex.co.kr (J.S.L.); chan.yoon@everex.co.kr (C.Y.); leonard@everex.co.kr (B.-H.K.); 3 Department of Biomedical Systems Informatics, Yonsei University College of Medicine, Seoul 03722, Republic of Korea; 4 Department of Psychiatry, Yonsei University College of Medicine, Seoul 03722, Republic of Korea; 5 Department of Rehabilitation Medicine, Hanyang University Guri Hospital, Hanyang University College of Medicine, Guri 11923, Republic of Korea

**Keywords:** gait analysis, pose estimation algorithm, markerless motion capture, marker based algorithm, verification

## Abstract

*Background and Objectives*: This study aimed to verify the validity of markerless gait analysis using both single-camera markerless system (S-ML) and multi-camera markerless system (M-ML) approaches by comparing them with a gold-standard marker-based system (MB). *Materials and Methods*: Sixteen healthy adults walked at their gait speed, and their gaits were simultaneously analyzed using three systems: S-ML, M-ML, and MB. Intraclass correlation coefficients were used to assess the reliability of the spatiotemporal parameters, and the root mean squared error (RMSE) was calculated to quantify the kinematic differences relative to the MB systems. *Results*: Both S-ML and M-ML demonstrated good-to-excellent reliability in spatiotemporal parameters, including step length, stance time, and gait speed, whereas stride length and swing time measured by S-ML revealed moderate reliability. In terms of joint kinematics, S-ML demonstrated a performance comparable to that of M-ML, particularly at the hip and knee in the sagittal plane. For certain parameters, such as knee abduction/adduction in the frontal plane, the S-ML demonstrated lower RMSE values than M-ML. In contrast, the ankle joint angles estimated using S-ML exhibited reduced agreement. *Conclusions*: In conclusion, markerless gait analysis can serve as an alternative to conventional gait analysis. However, certain parameters need to be improved.

## 1. Introduction

Gait analysis is crucial for the quantitative assessment of walking motion in rehabilitation medicine [1]. Gait analysis provides detailed kinematic and spatiotemporal data that are particularly valuable for characterizing movement abnormalities in patients with neurological and musculoskeletal disorders [2,3,4]. Such objective measurements can be used to understand the pathophysiology of the disease, tailor individualized treatments, and evaluate therapeutic outcomes [5,6,7].

Marker-based 3D motion capture systems are considered the gold standard for gait analysis because of their high measurement accuracy [8]. However, their use is limited by substantial costs, the requirement for a large dedicated laboratory space, and time-consuming preparatory procedures such as marker placement and system calibration [9]. In addition, the operation of these systems requires trained or specialized technicians [10,11]. These limitations significantly hindered the clinical use of gait analysis.

Recent advances in computer vision and deep learning technologies have enabled the development of markerless gait analysis as a viable alternative. Systems employing multiple 2D cameras in conjunction with human pose estimation algorithms have been introduced and are now commercially available [11,12,13]. These multi-camera systems are designed to reconstruct and calculate 3D joint angles by integrating data from multiple viewpoints. Various 2D human pose estimation algorithms based on deep learning, such as OpenPose, BlazePose or AlphaPose, facilitate the estimation of joint locations using a single-camera, allowing for the extraction of gait trajectories and joint kinematics, typically focusing on 2D planar analyses by projecting 3D movement onto the camera plane [11,14,15,16]. Markerless systems have several advantages, including the elimination of inconvenience and labor involved in marker placement, reduced setup time, and lower operational costs [11,17]. Moreover, these systems can be used outside the laboratory settings, enabling gait assessment in real-world environments. Accordingly, several studies have compared the accuracy of gait analysis between markerless and marker-based systems [8,10,12,13,18]. However, comparisons between 2D planar joint angles obtained from a single-camera and plane-specific angles derived from 3D motion capture data raise concerns regarding accuracy due to perspective-related errors, including camera alignment [19]. Nevertheless, in clinical practice, gait analysis is most often interpreted using sagittal and coronal plane angles derived from 3D motion capture rather than full 3D joint angle representations.

While previous studies have compared marker-based motion capture systems with either single-camera 2D or multi-camera markerless 3D gait analysis systems, few have simultaneously evaluated all three systems. This study aimed to assess the degree of agreement in spatiotemporal gait parameters and lower limb joint kinematics obtained from a single-camera markerless system (S-ML), a multi-camera markerless system (M-ML), and a marker-based 3D motion capture system (MB).

## 2. Materials and Methods

### 2.1. Participants and Ethics

This study recruited 16 healthy adults (9 males and 7 females; age: 22.94 ± 2.49 years; weight: 66 ± 14.19 kg; height: 169.25 ± 9.19 cm) without musculoskeletal or neurological disorders that could affect gait and without any history of orthopedic surgery in the past year. This study was approved by the institutional review board of Kookmin University (approval number: KMU-202301-HR-343; approval date: 6 March 2024). All participants were provided with verbal and written information pertaining to the study and all provided written informed consent to participate.

### 2.2. Gait Analysis

**Figure 1:**
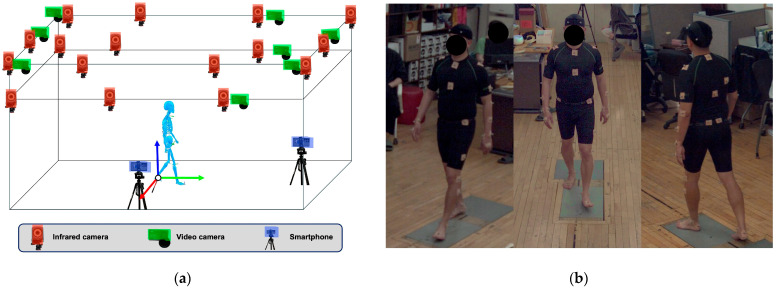
Overview of the experimental setup (**a**) 16 infrared cameras (red), 8 video cameras (green), and 2 smartphones (blue) were used, all of which were operated simultaneously during the gait; (**b**) Experimental measurement during gait.

Gait analysis was performed simultaneously using three systems (Figure 1):

(1). S-ML: smartphone-based single-camera markerless pose estimation system analyzing independent monocular views, in which two smartphones (Galaxy S20 FE, Samsung Electronics, Suwon, Republic of Korea) were positioned at the frontal and lateral sides of the walkway to capture frontal- and sagittal-plane gait data, respectively, with each camera processed independently as a single 2D view without multi-view integration or 3D reconstruction, consistent with its intended clinical use using a single handheld device [20];
(2). M-ML: A Theia3D system (Theia Markerless Inc., Kingston, ON, Canada) utilizing eight OptiTrack cameras (Optitrack Inc., Corvallis, OR, USA) demonstrating excellent validity and high inter-session reliability [21]; and
(3). MB: A Vicon system (Vicon Motion Systems Ltd., Oxford, UK) the gold standard with a consistently established error threshold of <5° across systematic reviews [22].

Participants were instructed to walk along a straight pathway, approximately 10 m in length, within the laboratory two times. For each participant, one representative gait cycle was extracted from each of the two trials (yielding two gait cycles per participant). These two cycles were treated as independent observations and pooled together for the subsequent analyses. Participants were instructed to walk a comfortable pace. The following variables were analyzed to compare the three systems: spatiotemporal parameters (gait speed, step length, stride length, stance time, and swing time) and lower–limb joint kinematics (hip flexion–extension and abduction–adduction, knee flexion–extension and abduction–adduction, and ankle dorsiflexion–plantarflexion).

**Figure 2:**
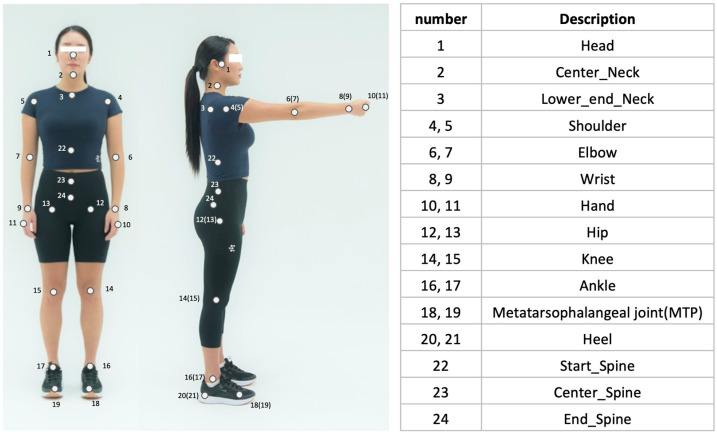
Key points of the pose estimation methods.

Gait analysis using the S-ML method was conducted as follows. First, video data were collected using two smartphone cameras positioned at the front and right side of the walkway (1920 × 1080 pixels, 60 frames/s). First, the YOLOv5 model was used for multi-person detection, with the main subject identified based on their proximity to the center (frontal view) and lateral boundary (lateral view) [23]. Subsequently, a single-person pose-estimation model, Griffin (EverEx, Seoul, Republic of Korea), was applied to extract 24 key anatomical points (Figure 2). We used ResNet-152 as the backbone to extract the visual features and was pretrained on a dataset of 87,666 images containing 121,502 annotated individuals performing various functional movement [24,25]. To ensure accuracy, we employed a post-processing weighted interpolation procedure based on confidence scores to refine low-certainty key points [26].

For kinematic analysis, joint angles were calculated from the corresponding optimal viewpoints: the lateral view for sagittal-plane angles (e.g., ankle dorsiflexion, hip/knee flexion) and the frontal view for frontal-plane angles (e.g., hip/knee abduction). This required the assumption that the optical axis of the lateral camera was perpendicular, and that of the frontal camera was parallel, to the line of progression. The predicted coordinates were used to compute joint angles, step timing, and step length. To measure the distance, we calibrate the pixel length based on the force plate (OR6-7-8000, AMTI Inc., Watertown, MA, USA) in the video, which had a known length of 46 cm. Except for spatial scaling based on the force plate, the S-ML system did not employ a formal geometric calibration procedure.

In M-ML, the video data from eight cameras were collected using a motion capture software (version 3.0.1, Optitrack, Natural Point Inc., Corvallis, OR, USA). The collected video data were processed using Theia 3D, which provided 4 × 4 transformation matrices for each segments [27]. Based on these matrices, lower limb joint angles and spatiotemporal parameters were calculated using Python v3.11.5 (Python Software Foundation, Wilmington, NC, USA). The M-ML system (Theia3D) used a checkerboard-based calibration procedure to define the 3D capture volume in accordance with the manufacturer’s guidelines.

Marker based data were collected using the Nexus software (version 2.12.1, Vicon Motion System Ltd., Oxford, UK). A total of 39 reflective markers were attached, and the Vicon Plug-in Gait Fullbody model was used. The participants assumed an anatomical posture before starting the walk to capture static motion, followed by the collection of gait data. Joint angles were calculated based on the collected marker data. Spatiotemporal variables were computed using Python. The MB system (Vicon) used a wand-based calibration procedure to define the 3D capture volume in accordance with the manufacturer’s guidelines.

These three systems were captured at a sampling rate of 60 Hz. For comparison across systems, a single gait cycle was defined based on the moment when the right foot first crossed the force plate. In the S-ML method, both frontal and lateral smartphone recordings were synchronized and calibrated using the audio signal generated at the time of acquisition. For inter-system comparisons across the S-ML, M-ML, and MB systems, additional temporal alignment was performed using the right heel-strike event. All metric outputs used for the analyses were derived from the calibrated views following this synchronization procedure.

### 2.3. Statistical Analysis

The accuracy of the spatiotemporal parameters was evaluated using the intraclass correlation coefficient (ICC), with ICC (2,1) applied to assess reliability. The root mean square error (RMSE) was calculated to analyze the joint angle parameters. The RMSE was used to quantify the differences in the time-series patterns of the gait cycle, as well as the maximum and minimum values throughout the gait cycle. The interpretation of ICC values followed established guidelines: values < 0.5 were considered to indicate poor reliability, those between 0.5 and 0.75 indicated moderate reliability, those between 0.75 and 0.9 indicated good reliability, and those > 0.9 were considered to indicate excellent reliability [28].

Sample size estimation was determined based on previous studies comparing markerless and marker-based gait analysis systems, which demonstrated good-to-excellent ICC [8,29,30]. The sample size of 16 participants was calculated using G*Power (version 3.1.9.7; α = 0.05, power = 0.80), assuming a large effect size (Cohen’s d = 1.0) based on joint angle discrepancies reported in prior studies.

## 3. Results

### 3.1. Spatiotemporal Parameters

Compared to the MB, both the S-ML and M-ML generally showed good-to-excellent reliability (Table 1). Step length was consistent across the three systems, with both S-ML and M-ML showing excellent reliability. Gait speed and stance time were also compared among the systems, with S-ML showing good reliability and the M-ML demonstrating excellent reliability. However, stride length and swing time measured using S-ML showed moderate reliability, whereas the M-ML exhibited excellent reliability for both parameters.

### 3.2. Kinematic Parameter

**Figure 3:**
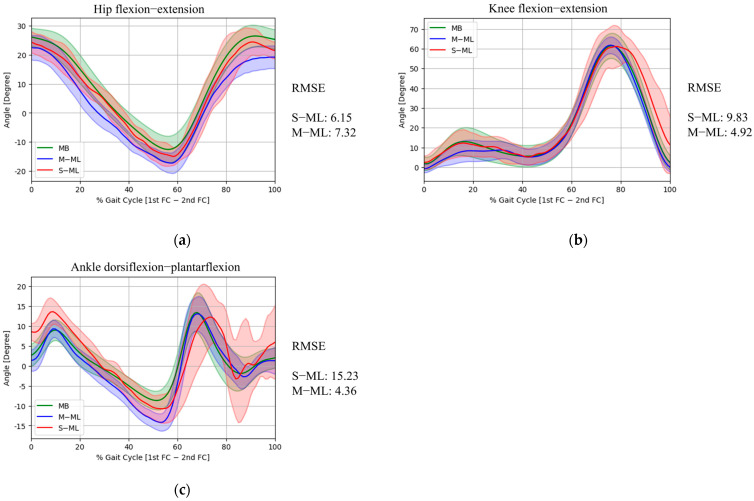
Comparison of sagittal-plane hip (**a**), knee (**b**), and ankle (**c**) joint kinematics across MB, M-ML, and S-ML during the gait cycle.

In the sagittal plane (Figure 3), the hip flexion/extension joint angles showed high agreement between the MB and S-ML, which had an RMSE 6.15° lower than that of the M-ML (7.32°). At peak hip flexion, S-ML measured 24.41 ± 4.55°, close to MB’s value of 26.44 ± 3.73°, with RMSE of 3.67°, which was also lower than that of M-ML (6.32°). For peak hip extension, S-ML was −14.94 ± 3.20°, while MB recorded −12.57 ± 4.56°, with a corresponding RMSE of 6.40°, slightly higher than M-ML (6.16°). The knee flexion and extension joint angles showed mixed results. The RMSE of the gait cycle was 9.83°, which was higher than that of M-ML (4.92°). Peak knee flexion was measured at 61.53 ± 6.36° by MB, 61.83 ± 4.23° by M-ML with RMSE of 4.52°, and 61.22 ± 10.59° by S-ML with RMSE of 8.52°. Peak knee extension was measured at 1.75 ± 3.33° by MB, −0.73 ± 2.05° by M-ML with RMSE of 4.65°, and 2.52 ± 3.37° by S-ML with RMSE of 3.56°. In contrast, ankle dorsiflexion and plantarflexion joint angles exhibited poor agreement between the S-ML and MB. In terms of the gait cycle, the RMSE of S-ML was 15.23°, which was higher than that of M-ML (4.36°). At peak ankle dorsiflexion, S-ML showed an RMSE of 7.03°, compared to 3.45° for M-ML. At peak ankle plantarflexion, the RMSE was 17.66° and 5.83° by S-ML and M-ML, respectively.

**Figure 4:**
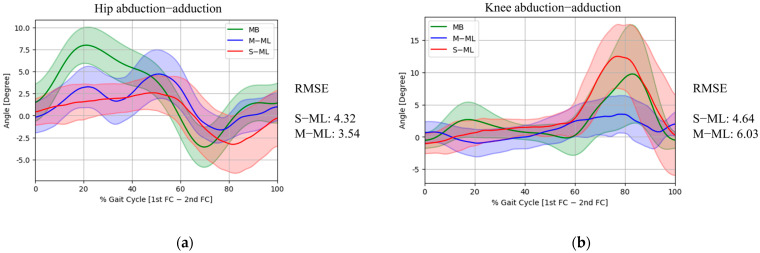
Comparison of frontal-plane hip (**a**) and knee (**b**) joint kinematics across MB, M-ML, and S-ML during the gait cycle.

In the frontal plane (Figure 4), both S-ML and M-ML at hip abduction tended to underestimate the magnitude of the joint angles compared with MB. Peak hip abduction was measured at 2.57 ± 1.95° by S-ML, compared to 7.99 ± 2.05° by MB, yielding an RMSE of 5.64°. The corresponding RMSE for M-ML was 3.79°. Peak hip adduction showed a similar value, with S-ML at −3.27 ± 3.26° and MB at −3.57 ± 2.28°, resulting in an RMSE of 2.92°. M-ML recorded a slightly lower value (−1.62 ± 1.40°), with an RMSE of 2.26°. In terms of the gait cycle, S-ML exhibited an RMSE of 4.32° for hip abduction/adduction, which was slightly higher than that of M-ML (3.54°). For peak knee abduction, the value measured by the S-ML (12.51 ± 4.96°) was closer to that of MB (9.78 ± 7.64°) than that of M-ML (3.53 ± 2.90°), and the corresponding RMSE of 6.89° (S-ML) was lower than that of M-ML (9.79°). Furthermore, across the gait cycle, the S-ML (4.64°) showed better RMSE results than the M-ML (6.03°) for knee abduction/adduction, suggesting that the S-ML followed the overall pattern of the knee abduction/adduction trajectory more closely.

## 4. Discussion

This study aimed to compare spatiotemporal gait parameters and lower-limb joint kinematics obtained from markerless gait analysis systems with those obtained from a marker-based gait analysis system. Our findings demonstrate that both S-ML and M-ML systems provide spatiotemporal parameters with good-to-excellent reliability compared to the MB gold standard, with the exception of stride length and swing time in S-ML, which showed moderate reliability. While sagittal plane kinematics for the hip and knee showed high agreement across all systems, discrepancies were more pronounced in the frontal plane and for sagittal kinematics at the ankle joint.

Spatiotemporal gait parameters are widely used in gait analysis owing to their high reproducibility and ability to provide essential information on gait performance, including functional mobility, gait symmetry, efficiency and balance [1]. In this study, parameters, including step length, stance time, and gait speed, measured using S-ML and M-ML revealed high accuracy and reliability compared with MB. These findings are consistent with those of previous studies that have demonstrated the potential of markerless motion capture systems to yield spatiotemporal data [8,12,13]. However, stride length and swing time measured via S-ML demonstrated only moderate reliability. For stride length, this may be attributed to increased spatial error accumulation over longer distances [20,31]. Stride length is the distance between consecutive heel strikes on the same foot and requires accurate long-distance measurements. Pose estimation algorithms tend to accumulate spatial errors over longer distances, and length errors are minimized when the participant is positioned at the center of the camera’s field of view [31]. This may also be related to the lack of formal 3D geometric calibration in the S-ML system, which relies on 2D pixel-to-meter scaling and cannot compensate for depth-dependent scaling changes during movement, in contrast to the MB and M-ML systems that employ 3D calibration, potentially increasing spatial uncertainty.

The moderate reliability of S-ML swing time may be attributed to the challenges in precise toe-off detection. Pose estimation methods often struggle to accurately identify small joints, particularly during movement, owing to key point misidentification [20,32]. Furthermore, shorter gait phases like the swing phase are inherently sensitive to temporal jitter. In the brief swing phase, even minor toe-off detection errors, possibly arising from monocular motion blur, produce significant relative errors. These discrepancies could be further intensified by differing algorithmic definitions of toe-off across systems. While both M-ML and MB determine toe-off using 3D spatial data, such as vertical trajectories and pelvis-relative displacement, S-ML identifies toe-off via 2D planar coordinate thresholds [33,34]. The absence of 3D depth in monocular views might make it more challenging to distinguish vertical ground clearance from horizontal progression.

Despite variability in reliability across parameters, the observed measurement differences relative to the MB system could be considered clinically acceptable based on commonly reported thresholds. Gait speed differences relative to the MB system for both S-ML and M-ML were well below the reported minimal clinically important difference of 0.1–0.2 m/s [35]. Step length differences relative to MB remained within published minimal detectable change ranges (2.1–6.7 cm) and below the minimal clinically important difference of approximately 3.6 cm reported in neurological populations [36,37]. Although stride length measured using S-ML demonstrated only moderate reliability, the observed difference relative to MB was within the range of minimal detectable change reported for step length. Similarly, differences in temporal parameters relative to MB, including stance time and swing time, remained below the reported MDC range of 3.2–4.2% of the gait cycle [37]. This included swing time measured using S-ML, despite its moderate reliability.

Joint kinematics analysis presents greater computational complexity than spatiotemporal analysis and is more sensitive to the accuracy of joint center detection [8,29]. Even minor errors in estimating the joint centers can result in substantial deviations in the joint angle, making kinematic parameters a more challenging measure of the accuracy of markerless motion analysis [29]. In this study, the S-ML and M-ML results were comparable to the MB results in the sagittal plane joint angles, particularly at the hip and knee. These findings are consistent with previous multi-camera markerless studies [12,13] as well as single-camera studies [20]. Similarly, a meta-analysis by Scatalini et al. [8] concluded that multi-camera markerless systems generally demonstrated higher reliability and accuracy in sagittal plane kinematics than in other planes.

In contrast, the ankle joint kinematics exhibited the greatest deviation, with the highest RMSE values among all joints. These findings are consistent with previous studies reporting lower reliability and accuracy of both single- and mult-camera markerless systems at distal joints, such as the ankle, owing to frequent occlusion, smaller movement amplitudes, and challenges in defining joint centers [8,11,17]. The ankle joint complex exhibits inherently complex 3D motion arising from coordinated interactions among the tibiotalar, talocalcaneal, and talocalcaneonavicular joints, which together produce tightly coupled tri-planar kinematics, including plantar–dorsiflexion, abduction–adduction, and inversion–eversion [38]. This kinematic complexity increases sensitivity to occlusion, small movement amplitudes, and errors in joint center estimation, all of which can substantially influence angle computation. In addition, the ankle involves substantial transverse-plane motion, which is difficult to fully capture from a single 2D viewpoint. Without depth information, as in the S-ML approach, variability in walking trajectories and subject–camera distance may increase uncertainty in distal joint measurements compared with 3D-calibrated systems.

In the frontal plane, the RMSE values of the hip angle during ambulation measured by both the S-ML and M-ML were relatively similar to those of the MB, although both the S-ML and M-ML tended to underestimate peak hip abduction compared to the MB. Regarding the knee angle, S-ML tended to overestimate knee abduction, whereas M-ML tended to underestimate it. These results are partially consistent with those of Wren et al. [13], who reported moderate reliability in frontal plane kinematics when comparing marker-based and multi-camera markerless systems. Similar limitations have also been reported in previous studies, indicating that both single- and multi-camera markerless systems may struggle to accurately capture complex multi-planar motions, particularly those involving rotational or lateral displacement [8,11,17]. For the S-ML system, these findings may be associated with two main factors: variability in pixel-to-meter scaling as participants move toward or away from the camera within the field of view, and limb foreshortening caused by sagittal-plane motion, both of which may affect frontal-plane joint angle [20,32,39]. Discrepancies in the M-ML system may arise from systematic differences between marker-based and markerless biomechanical model definitions. Marker-based systems define pelvic orientation using physical landmarks (e.g., ASIS and PSIS), typically resulting in a moderate anterior pelvic tilt, whereas the markerless M-ML system (Theia3D) relies on neural network–derived image features that tend to produce a more neutral pelvic reference frame [40]. These differences in pelvic coordinate system definition may lead to consistent offsets in frontal-plane kinematics across the gait cycle.

In this study, S-ML demonstrated agreement with M-ML for several parameters, although limitations persisted in length-related measures and ankle joint kinematics, suggesting the need for further refinement. However, because the proposed approach in this study is based on a single-camera 2D planar analysis, it inherently lacks true depth information and is therefore susceptible to perspective-related errors. In particular, as it does not utilize a depth camera, accurate depth estimation is not available [41] and measurement errors may occur when the subject moves closer to or farther away from the camera. Moreover, even in the sagittal view, joint position estimation may be affected when relevant body segments deviate from the center of the camera’s field of view. Recent studies have demonstrated that single consumer-grade depth cameras incorporating depth reconstruction can estimate 3D gait kinematics from 2D inputs [19,42]. In contrast, the present study focused on 2D projected joint angles derived from single-camera views rather than true 3D joint angles, which may partially explain the observed differences, especially when the subject was not perfectly aligned with the camera axis. This methodological difference may partly explain the discrepancies observed between S-ML and M-ML, which reconstructs full 3D kinematics using multiple cameras. However, these factors were not independently evaluated in this study, and further research is warranted to examine their individual contributions to measurement accuracy.

This study had several limitations. First, although the sample size was estimated to ensure sufficient statistical power, the final sample comprised only 16 participants. Despite the statistical justification, the limited number of participants may restrict the generalizability of the findings and the robustness of the conclusions. Second, all participants were young healthy adults without any neurological or musculoskeletal disorders. Therefore, it is uncertain whether these findings apply to older individuals or to patients with pathological gait patterns. Third, the experiment was conducted in a controlled laboratory environment, with participants wearing form-fitting clothing to minimize occlusion and facilitate joint detection. In real-world settings, individuals typically wear loose-fitting or varied types of clothing, which may interfere with markerless pose estimation and degrade measurement accuracy. Fourth, this study employed only one pose estimation algorithm (Griffin) and did not compare its performance with other commonly used open-source or commercial models [16]. Moreover, only Theia3D and Vicon were used as the M-ML and MB systems, respectively; although both are well-validated and widely adopted, the selection was limited by the systems available in our laboratory. To address these limitations, future studies should incorporate larger and more heterogeneous samples, simulate real-world test conditions, and conduct systematic comparisons across pose estimation models to strengthen the clinical utility and scalability of single-camera markerless motion capture systems.

## 5. Conclusions

This study evaluated single- and multi-camera markerless motion capture systems against a marker-based reference. Both systems demonstrated acceptable reliability in terms of spatiotemporal parameters and general agreement in joint kinematics, particularly in the sagittal plane. However, their accuracy is limited to certain parameters. These findings support the potential applicability of markerless systems for gait analysis, although further improvements are required.

## Figures and Tables

**Table 1:** Spatiotemporal parameters measured by MB, M-ML, and S-ML.

Parameter	S-ML	M-ML	MB	ICC (S-ML)	ICC (M-ML)
Gait speed (m/s)	1.090 ± 0.094	1.111 ± 0.092	1.091 ± 0.093	0.87	0.993
Step length (m)	0.617 ± 0.050	0.613 ± 0.046	0.609 ± 0.048	0.947	0.977
Stride length (m)	1.163 ± 0.109	1.223 ± 0.088	1.191 ± 0.091	0.658	0.981
Stance time (s)	0.733 ± 0.047	0.718 ± 0.040	0.725 ± 0.037	0.893	0.981
Swing time (s)	0.348 ± 0.036	0.386 ± 0.022	0.361 ± 0.021	0.722	0.922

Data are presented as mean ± standard deviation. S-ML, single-camera markerless system; M-ML, multi-camera markerless system; MB, marker-based motion capture system; ICC, intraclass correlation coefficient.

## Data Availability

The data presented in this study are available on request from the corresponding authors. The data are not publicly available due to privacy and ethical restrictions involving human participants, as well as commercial confidentiality agreements imposed by the co-researching company.

## Abbreviations

The following abbreviations are used in this manuscript:

S-ML	Single-camera markerless system
M-ML	Multi-camera markerless system
ICC	Intraclass correlation coefficient
RMSE	Root mean square error
3D	Three dimensional
2D	Two dimensional
